# Low incidence of paradoxical reductions in HDL-C levels in dyslipidemic patients treated with fenofibrate alone or in combination with ezetimibe or ezetimibe/simvastatin

**DOI:** 10.1186/1476-511X-10-212

**Published:** 2011-11-16

**Authors:** Michel Farnier, Qian Dong, Arvind Shah, Amy O Johnson-Levonas, Philippe Brudi

**Affiliations:** 1 Point Medical, 21000, Dijon, France; 2 Merck, Sharp & Dohme Corp., Whitehouse Station, New Jersey, USA

**Keywords:** high-density lipoprotein cholesterol, fibrates, dyslipidemia, ezetimibe, simvastatin

## Abstract

**Background:**

Fibrates have been reported to cause paradoxical decreases in HDL-C in certain patients.

**Design and methods:**

This post-hoc analysis explored the frequency/magnitude of HDL-C reductions in a pooled database of mixed dyslipidemic patients (LDL-C:3.4-5.7 mmol/L;TG:1.7-5.7 mmol/L) receiving placebo (PBO), fenofibrate (FENO), ezetimibe plus FENO (EZE+FENO), or EZE/simvastatin plus FENO (EZE/SIMVA+FENO) for 12 weeks.

**Results:**

PBO-treated patients had the highest incidence of HDL-C reductions from baseline (45%) compared with patients taking FENO (14%), EZE+FENO (9%), or EZE/SIMVA+FENO (9%). Reductions <30% reflected natural variability since the largest reduction in HDL-C approached 30% in the PBO group. Only 3 patients exhibited HDL-C reductions ≥30% (i.e., 2 patients in the FENO group and 1 in the EZE+FENO group). There were no differences in demographic/biochemical characteristics between patients with and without HDL-C reductions.

**Conclusions:**

The incidence of paradoxical HDL-C reductions was low in mixed dyslipidemic patients receiving FENO alone or combined with EZE or EZE/SIMVA.

**Trial registrations:**

Clinicaltrials.gov: NCT00092560 and NCT00092573

## Background

Plasma concentrations of high-density lipoprotein cholesterol (HDL-C) are inversely correlated with coronary heart disease (CHD) risk, even after adjusting for lipid and non-lipid risk factors [1]. This negative association is maintained even at very low levels of low-density lipoprotein cholesterol (LDL-C) [2]. As a consequence, increasing HDL-C has emerged as an attractive tool for preventing cardiovascular events. Moreover the presence of atherogenic dyslipidemia, which is characterized by low HDL-C and elevated fasting and postprandial triglyceride (TG) levels, contributes strongly to CHD risk even when LDL-C is well controlled [3,4]. The atherogenic dyslipidemia phenotype is usually observed in patients with mixed dyslipidemia, type 2 diabetes and/or metabolic syndrome [5]. Beyond lifestyle approaches, fibrates are one of the available strategies to treat atherogenic dyslipidemia and to prevent CHD [5,6].

Fibrates are agonists of peroxisome proliferator-activated receptor-α (PPAR-α). By activation of PPRA-α, fibrates impact multiple pathways of lipid metabolism while also exerting pleiotropic effects through the regulation of genes influencing vascular inflammation and thrombogenesis [7]. Several large-scale trials of fibrate therapy have been completed with conflicting results on cardiovascular outcomes [8-11]. However, recent meta-analyses have shown that fibrates can reduce the risk of cardiovascular events predominantly through the prevention of coronary events [12,13]. Moreover post-hoc analyses of several of these fibrate trials and meta-analyses provided consistent evidence of a clinical benefit in the subgroup of patients with atherogenic dyslipidemia [5,14].

Fibrates modulate the atherogenic lipid profile by concomitantly lowering TG levels (up to 50%) and raising HDL-C levels (up to 10%-15%) [15]. These effects differ among fibrates, and the long-term HDL-raising effect is less (<5%) in people with type 2 diabetes [4,9]. In a recent meta-analysis, the mean HDL-C increase observed following fenofibrate (FENO) therapy was 10.2% [12]. Several recent reports have suggested that fibrates, particularly FENO, may cause paradoxical reductions in HDL-C levels in certain patient populations, such as patients with type 2 diabetes, elevated or reduced pre-treatment HDL-C levels, and following concomitant use with statins and/or other medications (e.g., thiazolidinediones) [16,17]. However, there is wide variability in the documented literature regarding the frequency and magnitude of paradoxical HDL-C reductions seen in association with FENO treatment. The absence of placebo-treated patients in these studies precluded an assessment of whether these paradoxical HDL-C reductions were in part due to the natural variability in HDL-C changes over time and/or differences in measurement techniques between studies.

The purpose of this post-hoc analysis was to explore the frequency and magnitude of paradoxical HDL-C reductions during FENO therapy in a large pooled database of mixed dyslipidemic patients receiving placebo, FENO monotherapy, or FENO in combination with ezetimibe (EZE) or EZE/simvastatin (SIMVA) treatments. This pooled analysis offers several advantages over prior studies, including the large number of FENO-treated patients (*n *= 731) contributing to the analysis and the presence of a placebo group, which served as a control for the natural variability in HDL-C changes over time in this population of mixed dyslipidemic patients. Moreover, this analysis gives the opportunity to obtain information on this paradoxical effect when FENO is combined with other lipid-lowering drugs.

## Results

Of 854 patients in the pooled database with paired HDL-C values at baseline and study end, a total of 139 (16%) patients in all groups had reductions from baseline in HDL-C at study endpoint (Table 1). A similar proportion of PBO-treated patients experienced HDL-C increases and HDL-C decreases at study endpoint (i.e., 52% vs. 45%, respectively). The overwhelming majority of patients in the active treatment groups experienced increases from baseline in HDL-C (i.e., 84% in FENO group and 89% in each the EZE + FENO and EZE/SIMVA + FENO groups). PBO-treated patients had the highest incidence of HDL-C reductions from baseline (45%) compared with patients taking active treatment with FENO alone (14%), EZE + FENO (9%), or EZE/SIMVA + FENO (9%). The incidence of paradoxical HDL-C reductions was lowest in the EZE + FENO and EZE/SIMVA + FENO groups. A small and similar proportion of patients experienced no change from baseline in HDL-C across the treatment groups. As a result, patients who experienced increases or no changes from baseline in HDL-C were pooled together within each treatment group for the purpose of all categorical analyses.

**Table 1:** Numbers (%) of patients with increases, no change, or decreases from baseline in HDL-C at endpoint presented by treatment group

	HDL-C increase and no change		
		
Treatment Group	HDL-C increase n (%)	No change n (%)	HDL-C decrease n (%)
PBO (n = 123)	64 (52.0)	4 (3.3)	55 (44.7)
FENO (n = 368)	309 (84.0)	8 (2.2)	51 (13.9)
EZE+FENO (n = 183)	163 (89.1)	3 (1.6)	17 (9.3)
EZE/SIMVA+FENO (n = 180)	161 (89.4)	3 (1.7)	16 (8.9)

EZE = ezetimibe; FENO = fenofibrate; PBO = placebo; SIMVA = simvastatin

**Figure 1:**
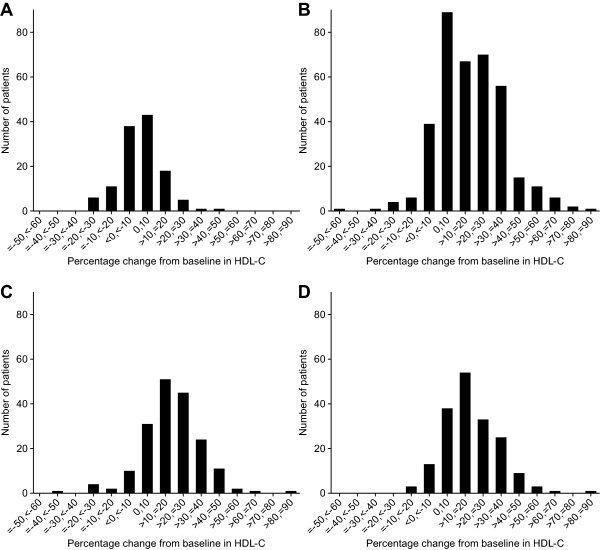
**Distribution in mean percent**. Distribution in mean percent changes from baseline in HDL-C for patients in the placebo group (n = 123) (**A**); fenofibrate 160 mg group (n = 368) (**B**); ezetimibe 10 mg plus fenofibrate 160 mg group (n = 183) (**C**); and ezetimibe/simvastatin 10/20 mg plus fenofibrate 160 mg group (n = 180) (**D**)

Distribution graphs for percent change from baseline in HDL-C at study endpoint were examined for each of the individual treatment groups (Figure 1). For the PBO group, patients had increases ranging from >0% to 50% and decreases from baseline ranging from >0% to approximately 30% at study endpoint, with the majority of the increases and decreases from baseline ranging from 0% to approximately 20% (Figure 1A). In comparison, patients in the FENO, FENO + EZE, and FENO + EZE/SIMVA groups had increases ranging from >0% to 90% and decreases from baseline ranging from >0% to 60% at study endpoint (Figure 1B-1D). Most patients receiving FENO experienced increases in HDL-C ranging from >0% to 40% irrespective of whether FENO was administered alone or in combination with EZE or EZE/SIMVA (Figure 1B-1D). The majority of patients in the FENO, EZE+FENO, and EZE/SIMVA+FENO groups experienced small reductions in HDL-C on the order of 0% to approximately 20% from baseline (Figure 1B-1D).

The baseline demographics and lipid characteristics were generally similar for the cohorts of patients experiencing reductions and increases/no change in HDL-C at study endpoint (Table 2). In general, patients with HDL-C reductions at study end had slightly higher HDL-C and Apo AI values and slightly lower non-HDL-C and TG levels at baseline (Table 2).

**Table 2:** Demographics and baseline characteristics of patients with increases/no change or decreases from baseline in HDL-C at endpoint

	HDL-C increase and no change	HDL-C decrease	All patients
**Demographic Parameters**			
Mean age ± SD, years (n)	54.6 ± 10.3 (715)	52.6 ± 10.8 (139)	54.3 ± 10.4 (854)
<65 years, n (%)	578 (80.8%)	122 (87.8%)	700 (82.0%)
≥65 years, n (%)	137 (19.2%)	17 (12.2%)	154 (18.0%)
Gender, n (%)			
Female	340 (47.6%)	57 (47.6%)	397 (46.5%)
Male	375 (52.4%)	82 (59.0%)	457 (53.5%)
Race category, n (%)			
Caucasian	561 (78.5%)	111 (79.9%)	672 (78.7%)
Non-Caucasian	154 (21.5%)	28 (20.1%)	182 (21.3%)
History of diabetes, n (%)	86 (12.0%)	20 (14.4%)	106 (12.4%)
**Baseline Parameters, mmol/L (n)**			
Mean HDL ± SD	1.1 ± 0.2 (715)	1.2 ± 0.3 (139)	1.1 ± 0.2 (854)
Mean Apo AI ± SD (g/L)	1.5 ± 0.2 (706)	1.6 ± 0.4 (134)	1.5 ± 0.3 (840)
Mean LDL-C ± SD	4.2 ± 0.7 (715)	4.2 ± 0.7 (139)	4.2 ± 0.7 (854)
Mean Apo B ± SD (g/L)	1.7 ± 0.2 (706)	1.6 ± 0.3 (134)	1.6 ± 0.2 (840)
Mean Non-HDL-C ± SD	5.6 ± 0.8 (715)	5.4 ± 0.8 (139)	5.6 ± 0.8 (854)
Mean TC ± SD	6.7 ± 0.9 (715)	6.6 ± 0.8 (139)	6.7 ± 0.8 (854)
Median TG ± SD	2.9 ± 1.0 (715)	2.7 ± 1.1 (139)	2.9 ± 1.0 (854)

Apo = apolipoprotein; LDL-C = low-density lipoprotein cholesterol; HDL-C = high-density lipoprotein cholesterol; SD = standard deviation; TC = total cholesterol; TG = triglycerides

**Figure 2:**
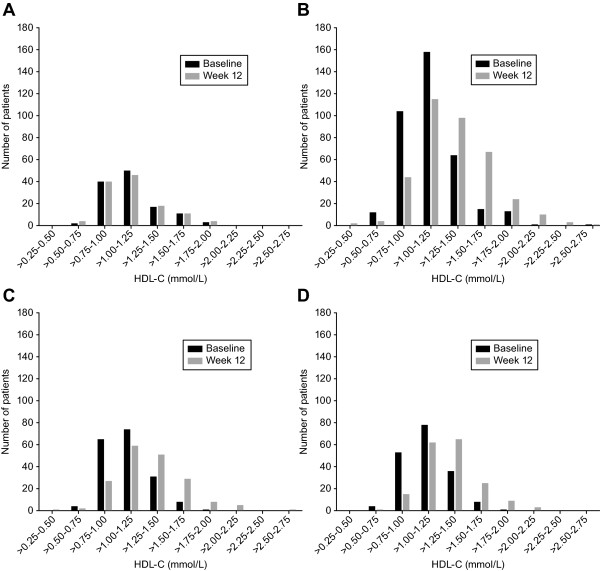
**Distribution of HDL-C values**. Distribution of HDL-C values (mmol/L) at baseline and study endpoint for patients in the placebo group (n = 123) (**A**); fenofibrate 160 mg group (n = 368) (**B**); ezetimibe 10 mg plus fenofibrate 160 mg group (n = 183) (**C**); and ezetimibe/simvastatin 10/20 mg plus fenofibrate 160 mg group (n = 180) (**D**)

The distributions in HDL-C values at baseline and study endpoint were plotted for each of the individual treatment groups (Figure 2). The distributions of HDL-C levels were nearly identical at baseline and study endpoint for patients receiving PBO (Figure 2A). In contrast, there was a shift to the right (i.e., shift toward higher HDL-C levels) in the distribution of HDL-C values at endpoint relative to baseline in patients receiving FENO, EZE+FENO, and EZE/SIMVA + FENO (Figure 2B-2D).

Modest reductions from baseline <30% were considered to reflect natural variability in the HDL-C measurement since the largest observed reduction from baseline in HDL-C approached 30% in the PBO group (Figure 1A). In general, the incidences of HDL-C reductions <30% were similar across the PBO, FENO, EZE + FENO and EZE/SIMVA + FENO groups (Table 3). Only three patients exhibited reductions in HDL-C of ≥30% in magnitude (i.e., 2 patients in the FENO group and 1 patient in the EZE + FENO group; Table 3). The actual observed HDL-C reductions for these three patients were 57%, 49%, and 30% (Table 4). Two of the three patients had Apo AI reductions that were generally commensurate with the observed HDL-C reductions whereas one patient with an HDL-C reduction of 30% had an Apo AI reduction of only 2.4%. None of these patients were taking concomitant medications with a known potential to modify HDL-C levels.

**Table 3:** Proportions of patients (%) in each treatment group with reductions from baseline in HDL-C < and ≥30% at endpoint

Treatment Group	<30%	≥30%
PBO	55/55 (100)	0
FENO	49/51 (96)	2/51 (4)
EZE+FENO	16/17 (94)	1/17 (6)
EZE/SIMVA+FENO	16/16 (100)	0

EZE = ezetimibe; FENO = fenofibrate; PBO = placebo; SIMVA = simvastatin

**Table 4:** Detailed listing of concomitant medications and patient characteristics for the 3 patients with HDL-C reductions ≤30% at study endpoint

					HDL-C (mmol/L)		Triglycerides (mmol/L)		Apo AI (g/L)	
					
Treatment	Concomitant Therapy	Age (yr)	Sex	BMI	Baseline	Endpoint (% change)	Baseline	Endpoint (% change)	Baseline	Endpoint (% change)
Reduction between 30%-40%										
Feno	None	36	Male	<30	1.04	0.73 (-30.00)	3.48	3.59 (3.25)	1.26	1.23 (-2.38)

Reduction between >40%-50%										
EZE+Feno	celecoxib	43	Female	<30	0.85	0.44 (-48.48)	3.44	2.25 (-34.65)	1.18	0.53 (-55.1)
	acetaminophen									
	clonazepam									
	chlorthalidone									
	propanolol HCl									

Reduction over 50%										
Feno	acetaminophen acetylcysteine	52	Male	<30	1.07	0.47 (-56.63)	1.94	3.27 (68.02)	1.47	0.54 (-63.3)
	cetrimide+naphazoline nitrate+phenylephrine hydrochloride+prednisolone									
	fenspiride hydrochloride									
	bacitracin zinc + tixocortol pivalate									
	oxomemazine									
	ibuprofen									
	telithromycin									
	guaifenesin+oxomemazine+sodium benzoate									
	allopurinol									
	niaouli oil+quinine benzoate+thiamine HCl									
	losartan potassium									

BMI = body mass index; EZE = ezetimibe; Feno = fenofibrate

## Methods

This was a pooled post-hoc analysis of data from two multicenter, international, randomized, double-blind, placebo-controlled, parallel-group studies (Merck Protocol Numbers MK-0653A-036 and MK-0653-071; Clinical registrations: NCT#00092560 and NCT#00092573). Eligible patients included men and women 18 to 79 years of age with mixed hyperlipidemia and no CHD, CHD-equivalent disease (except for type 2 diabetes), or CHD risk score >20% as defined by the National Cholesterol Education Program Adult Treatment Program III (NCEP ATP III).

The studies were conducted in accordance with principles of Good Clinical Practice, and the protocols and procedures were approved by an Ethics Review Committee (ERC) or Institutional Review Board (IRB) for each participating study site. Merck's approach to the conduct of clinical trials is in accordance with the ethical principles that have their origin in the Declaration of Helsinki, and that are consistent with Good Clinical Practice and the applicable regulatory requirement(s). Prior to any study-related procedures, all planned procedures and inherent risks were reviewed with each patient and/or their representative, and all patients provided written informed consent to participate in the study.

After a drug washout and a PBO run-in period with dietary and life style counseling, patients were allowed to enter the studies if their plasma lipid concentrations met the following criteria for randomization: LDL-C 3.4 to 5.7 mmol/L and TG 1.7 (2.3 for EZE/SIMVA + FENO study) to 5.7 mmol/L inclusive. Patients with type 2 diabetes were limited to those with LDL-C 2.6 to 4.7 mmol/L, inclusive.

In the first study, qualifying patients were randomized to one of the following daily treatments for 12 weeks: PBO (n = 64); FENO 160 mg (*n *= 189); EZE 10 mg (*n *= 187); or EZE 10 mg +FENO 160 mg (*n *= 185). In the second study, qualifying patients were randomized to one of the following daily treatments for 12 weeks: PBO (*n *= 60); FENO 160 mg (*n *= 184); EZE/SIMVA, *n *= 184); EZE/SIMVA 10/20 mg + FENO 160 mg (*n *= 183)

For the purpose of this analysis, available data from the PBO (*n *= 123) and FENO (n = 368) arms were combined across studies, and data from the PBO, FENO, EZE+FENO, and EZE/SIMVA+FENO arms were included in the analysis.

All lipid and apolipoprotein (Apo) analyses for the two studies were performed using the same validated assay methods at the same central laboratory (either PPD facilities, Medical Research Laboratory, Highland Heights, Kentucky, USA or Zaventem, Belgium).

This analysis examined the frequency and magnitude of HDL-C changes from baseline in each of the four treatment groups. The numbers and percentages of patients with HDL-C decrease, no change, and increase at the end of treatment versus baseline were tabulated by treatment arms. The proportions of patients with no change in HDL-C at study end were low and similar across the treatment groups. As a result, the frequency of HDL-C increases and frequency of no change from baseline were summated together for the purpose of all categorical analyses. The distributions of the percentage change from baseline in HDL-C values at study endpoint as well as HDL-C values pre- and post-treatment were displayed as histograms for each of the treatment groups.

Descriptive statistics of the baseline characteristics and plasma lipid profiles for patients with HDL reductions versus those with HDL increases (or no change) were summarized. The age, gender, body mass index (BMI), concomitant medications, and baseline/endpoint HDL-C, TG, and Apo AI values were evaluated for every patient receiving active treatment (i.e., FENO, EZE + FENO or EZE/SIMVA + FENO) who had HDL-C reductions ≥30%. The cut point of ≥30% was selected since the maximum percentage change from baseline in HDL-C values for individual patients observed in the placebo group approached 30%.

## Discussion

This post-hoc pooled analysis of two previously published randomized, double-blind, placebo-controlled studies examined the incidence of paradoxical reductions from baseline in HDL-C following treatment with FENO (i.e., FENO administered alone or in combination with EZE or EZE/SIMVA) or PBO for 12 weeks in a large population of patients (N = 854) with mixed dyslipidemia. The present analysis was undertaken to evaluate whether paradoxical HDL-C reductions following FENO treatment is a common or a rare occurrence. Prior studies have examined this question and arrived at very different conclusions. A retrospective analysis of lipid data from 94 patients taking FENO (i.e., micronized FENO 200 mg or supra-micronized FENO 160 mg) for 8 to 12 weeks showed that reductions in HDL-C occurred in almost half of the study population (i.e., 46%)[18]. Nine patients (9.6%) in that study experienced large magnitude HDL-C reductions of >50%. The reductions in HDL-C appeared to occur more frequently in patients with low pre-treatment HDL-C levels (<0.9 mmol/l). A subsequent retrospective analysis of lipid data from 581 patients reported that the incidence of paradoxical HDL-C reductions was a relatively uncommon phenomenon occurring in only 15% of the study population, with overall modest decreases from baseline in HDL-C (<50%) [19].

Beyond these analysis, a number of cases have been reported in the literature in which fibrates, particularly FENO and ciprofibrate, have been associated with paradoxical reductions in HDL-C levels[16,20,21]. These paradoxical decreases seem more frequent when a fibrate is combined with thiazolidinediones [17,22-26]. Other reports suggest a higher risk of paradoxical reductions in HDL-C during fibrate/statin combination therapy [16,27] and for patients with diabetes [27]. It is important to confirm or disprove a specific risk for diabetic patients treated with a statin since this statin treatment is recommended for almost all patients with type 2 diabetes [28,29] and since a combination with FENO seems particularly useful for patients with atherogenic dyslipidemia [4,14].

In the current pooled analysis of mixed dyslipidemic patients, the overall incidence of paradoxical HDL-C reductions was approximately 11.5% (84/731) across the pooled FENO (i.e., FENO, EZE + FENO and EZE/SIMVA + FENO) treatment groups compared with 44.7% (55/123) in PBO-treated patients.

There were no ascertainable differences in the baseline demographics between patients who experienced HDL-C reductions and those who experienced increases/no change from baseline. In general, patients with HDL-C decreases had slightly higher HDL-C and Apo AI values and slightly lower non-HDL-C and TG levels at baseline. This finding suggests that a regression to the mean phenomenon may be at least in part responsible for the observed reductions in HDL-C seen with PBO and FENO treatment in this study. The HDL-C levels in a patient with high pre-treatment HDL-C values might be expected to decrease due to a natural tendency to regress to the population mean.

Modest reductions from baseline <30% were considered to reflect natural variability in the HDL-C measurement [30] since the largest observed reduction from baseline in HDL-C approached 30% in the PBO group. The vast majority of FENO-treated patients (96%) had HDL-C reductions from baseline <30% in magnitude. Only three patients in the FENO-treated groups (2 patients taking FENO and 1 patients taking EZE+FENO) had HDL-C reductions from baseline of 30% or more. For the first patient, the decrease in HDL-C was not associated with a reduction in the Apo AI level. This finding also suggests that the observed reduction in HDL-C for this patient was due to the variability in the dosage of HDL-C[30]. For the two other patients, the observed reductions in Apo AI (-55% and -63%) were commensurate with the observed magnitude reduction in HDL-C (-49% and -57%, respectively). There were no notable differences in the demographic or baseline characteristics of the three patients with HDL-C reductions ≥30% compared with other patients included in this analysis. Furthermore, none of these patients were taking prescription and/or non-prescription medications with a known propensity to modify HDL-C levels.

The mechanism of action(s) underlying the paradoxical decreases in HDL-C seen following fibrate treatment remains unresolved. A pharmacogenetic association between the Apo AI/C3/A4/A5 gene cluster and lipid responses to fenofibrate has been described[31]. But the reported results do not support a role for this gene cluster in large magnitude HDL-C reductions. The corresponding decrease in Apo AI seen with HDL-C reductions in the current study suggests a role for Apo AI metabolism. A prior study evaluated the metabolism of Apo AI and Apo AII in a single patient with a paradoxical reduction in HDL-C due to ciprofibrate[32]. This study found an increased production rate of Apo AI and decreased residence time at baseline, and a further decreased residence time during ciprofibrate treatment while production rate remained increased. A putative interaction with the peroxisome proliferator response elements in the promoter for the Apo AI gene cannot be excluded.

It is worth noting that treatment with FENO, EZE + FENO, and EZE/SIMVA + FENO did produce expected increases from baseline in HDL-C. Most patients treated with FENO had increases from baseline in the 10% to 50% range, demonstrating the effectiveness of FENO treatment in raising HDL-C levels. In contrast, relatively few patients receiving PBO (i.e., 52%) had increases from baseline in HDL-C versus 84%, 89%, and 89% of the patients in the PBO, FENO, EZE + FENO and EZE/SIMVA + FENO groups, respectively. The magnitudes of the increases in HDL-C were considerably smaller in the PBO-treated patients than FENO-treated patients.

Taken together, the results of our findings do not substantiate findings in a previous publication that reported a high incidence (46%) of large magnitude reductions in HDL-C (>50%) in patients receiving FENO treatment[18]. In contrast, the results of this current large, pooled analysis demonstrate that the incidence of HDL-C reductions occurred less frequently in FENO-treated patients than in PBO-treated patients (12% versus 45%, respectively). Furthermore, the vast majority of HDL-C reductions seen in FENO-treated patients were modest in magnitude (<30%) and similar to those seen among PBO-treated patients. Only two patients in the current study had HDL-C reductions >50%, with concomitant and similar decreases in Apo AI. These patients did not have a diagnosis of diabetes and were receiving statin treatment. In this analysis, there were no specific patient characteristics predictive of the risk of HDL-C decrease.

In conclusion, the overall incidence of paradoxical HDL-C reductions was low in this pooled analysis of mixed dyslipidemic patients receiving FENO alone or combined with EZE or EZE/SIMVA.

## Abbreviations

Apo: apolipoprotein; BMI: body mass index; CHD: coronary heart disease; EZE: ezetimibe; FENO: fenofibrate; HDL-C: high-density lipoprotein cholesterol; LDL-C: low-density lipoprotein cholesterol; PBO: placebo; SIMVA: simvastatin; TG: triglycerides

## Competing interests

Authors Dong, Shah, Johnson-Levonas, and Brudi are current or former employees of Merck, Sharp & Dohme Corp, a subsidiary of Merck & Co., Inc., Whitehouse Station, NJ and may own stock or hold stock options in the company. Author Farnier has received research grant support, honoraria, and served as consultant and/or advisory board member for several companies, including Merck and Merck/Schering-Plough Pharmaceuticals. The current affiliation of author Q. Dong is Celgene Corporation, Summit, NJ.

Funding for the original study and the post-hoc analysis described here were provided by Merck & Co. Inc., Whitehouse Station, NJ. The sponsor had no role in the collection, analysis and interpretation of the data, or writing of the report and was not involved in the decision to submit the manuscript.

## Authors' contributions

All authors provided substantial contributions to the concept and design of the study (and the present post-hoc analysis), analysis and interpretation of data, and drafting/revision of the manuscript. All authors approved the final version for submission.
